# Decreased natural organic matter in water distribution decreases nitrite formation in non-disinfected conditions, via enhanced nitrite oxidation

**DOI:** 10.1016/j.wroa.2020.100069

**Published:** 2020-09-30

**Authors:** Pirjo-Liisa Rantanen, Minna M. Keinänen-Toivola, Merja Ahonen, Alejandro González-Martínez, Ilkka Mellin, Riku Vahala

**Affiliations:** a Department of Built Environment, Aalto University, PO Box 15200, FI-00076, Aalto, Finland; b Faculty of Technology, Satakunta University of Applied Sciences, PO Box, 1001, FI-28101, Pori, Finland; c Department of Microbiology, University of Granada, Campus Universitario de Cartuja, 18071, Granada, Spain; d Department of Mathematics and Systems Analysis, Aalto University, PO Box 11100, FI-00076, Aalto, Finland

**Keywords:** Nitrite, Nitrification, Natural organic matter, Water distribution systems, Non-disinfected conditions

## Abstract

Nitrite in drinking water is a potentially harmful substance for humans, and controlling nitrite formation in drinking water distribution systems (DWDSs) is highly important. The effect of natural organic matter (NOM) on the formation of nitrite in simulated distribution systems was studied. The objective was to inspect how a reduced NOM concentration affected nitrite development via nitrification, separated from the effects of disinfection. We observed that nitrite formation was noticeably sensitive to the changes in the NOM concentrations. Nitrite declined with reduced NOM (TOC 1.0 mg L^-1^) but increased with the normal NOM concentration of tap water (TOC 1.6 mg L^-1^). Ammonium oxidation was not altered by the reduced NOM, however, nitrite oxidation was enhanced significantly according to the pseudo-first order reaction rate model interpretation. The enhanced nitrite oxidation was observed with both ammonium and nitrite as the initial nitrogen source. The theoretical maximum nitrite concentrations were higher with the normal concentration of NOM than with reduced NOM. The results suggest that the role of nitrite oxidation may be quite important in nitrite formation in DWDSs and worth further studies. As a practical result, our study supported enhanced NOM removal in non-disinfected DWDSs.

## Highlights

• Nitrite build-up was prevented by reduced levels of natural organic matter.
• The reason for the decreased nitrite formation was enhanced nitrite oxidation.
• Ammonium oxidation was not affected by changes in natural organic matter.

## Graphical abstract

**Figure:**
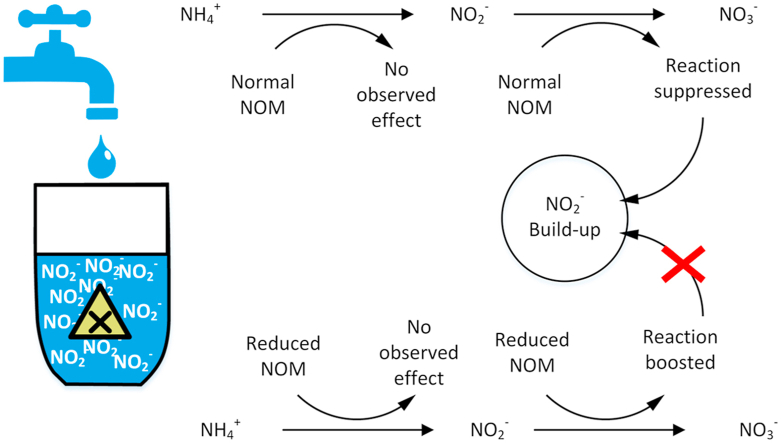

## 1 Introduction

Nitrification and nitrite formation are unwanted, but widely observed occurrences in drinking water distribution with water containing free ammonium or an ammonium source. Autotrophic nitrification is a two-phase microbiological reaction: first, ammonium is oxidized into nitrite (Equation (1)) and, second, nitrite is oxidized into nitrate (Equation (2)) as follows:

(1) 2 NH_4_^+^ + 3 O_2_ → 2 NO_2_^‒^ + 2 H_2_O + 4 H^+^

(2) 2 NO_2_^‒^ + O_2_ → 2 NO_3_^‒^.

Equation (1) is called *nitritation* and Equation (2) is called *nitratation*. Nitrite is formed as an intermediate product of these two reactions. Nitritation occurs by ammonia oxidizing bacteria (AOB), and nitratation by nitrite oxidizing bacteria (NOB). The most common type of AOB is *Nitrosomonas* sp., and the most common type of NOB is *Nitrospira* sp. The AOB and the NOB are aerobic chemolithoautotrophic organisms that consume carbon dioxide and do not need organic matter for their growth.

In general, organic matter suppresses nitrification. This effect is largely due to competition between the fast-growing, organic matter–consuming heterotrophic bacteria and the slowly growing AOB and NOB, which lose in this competition for substrate, oxygen, nutrients, and space. Furthermore, AOB and NOB are forced deeper into biofilms by rapid heterotrophic growth, which leads to a greater mass-transport resistance (Furumai and Rittmann, 1994; Rittmann and Manem, 1992).

In drinking water, natural organic matter (NOM) originates from the surface or groundwater used as raw water in water treatment. While most of the particulate NOM is removed during drinking water production, soluble NOM remains in the distributed water (Nissinen et al., 2001). In drinking water distribution systems (DWDSs), NOM has been observed to have opposing effects on nitrification. In most research in the USA, high NOM concentrations were associated with increased nitrite formation (Harrington et al., 2002; Wilczak et al., 2003; Yang et al., 2008b; Zhang et al., 2010). On the other hand, decreasing NOM by granular activated carbon (GAC) filtration has induced increased nitrite concentrations in the USA and Finland (Skadsen, 1993; Vahala et al., 1999). However, the research of Skadsen et al. included the addition of monochloramine prior the GAC filters, which caused nitrification in the biofilm of the GAC filter. Thus, the DWDS was inoculated with AOB and NOB from the produced water. Furthermore, in Canada, no correlation between NOM and nitrite concentrations was found in two DWDSs (Scott et al., 2015). Huang et al. (2016) noticed that the dissolved organic matter (DOM) in the molecular size range of 200–500 g mol^-1^ correlated positively with oxidized nitrogen and with active bacterial cells in a DWDS disinfected with monochloramine.

Nitrite can be formed in DWDSs from the ammonium in the raw water. For example, in China ammonium concentrations of 0.1–3 mg L^-1^ have been reported, and the highest values may reach more than 10 mg L^-1^ (Zhang et al., 2019). Also, in Denmark, the groundwater used as drinking water contained ammonium and nitrite (Schullehner et al., 2017). Moreover, a common disinfectant, monochloramine, provides ammonium when decomposed, thus promoting nitrite formation (Harrington et al., 2002; Wilczak et al., 1996; Wolfe et al., 1990). It has also been observed that a significant share of monochloramine itself can be directly metabolized into nitrite (Maestre et al., 2016; Wahman et al., 2016). In DWDSs, nitrification can be prevented by other disinfection chemicals, for example, free chlorine (Rosenfeldt et al., 2009). However, chlorination enhances the formation of disinfection by-products (DBPs). According to the European directive 98/83/EC, the maximum admissible concentrations of nitrite, nitrate and ammonium in drinking water are 0.5 mg L^-1^, 50 mg L^-1^, and 0.5 mg L^-1^, respectively (European Council, 1998).

The effect of NOM on nitrite formation in DWDSs with disinfection chemicals, especially monochloramine, has been inspected in several studies, as described above. However, studies without disinfection chemicals are rare. Thus, to determine the effects of NOM, separated from the effects of disinfection chemicals, laboratory-scale tests were organized in this study. Non-disinfected DWDSs are common, for example, in Europe (Lipponen et al., 2002; Schullehner et al., 2017; Waak et al., 2018). The objective of the study was to evaluate how the nitrite concentrations are affected by reduced concentrations of NOM in conditions relevant to water distribution. Furthermore, the focus was to understand the roles of both nitritation and nitratation.

## 2 Materials and methods

### 2.1 A description of the simulated distribution systems

**Fig. 1:**
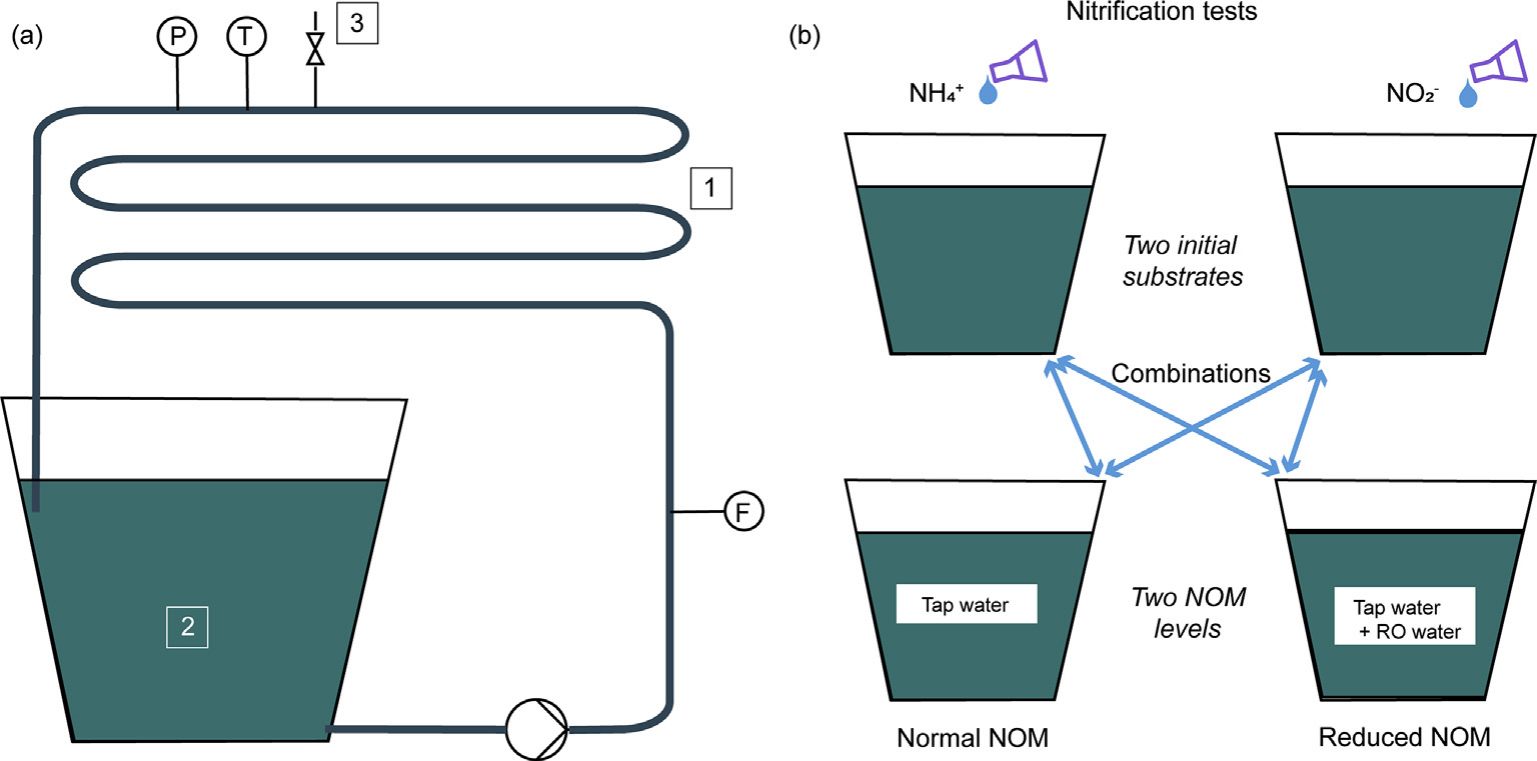
The figure depicts (a) a schematic diagram of the SDSs where 1 = a pipe loop of 22 m, 2 = a covered tank with a water volume of 30 L, 3 = a vent valve, F = a water flow meter, P = a pressure gauge, T = a thermometer; two similar SDSs (SDS1 and SDS2) were used in the study; and (b) a diagram of the performed tests; nitrification tests were executed with ammonium and nitrite as initial substrates and two levels of NOM were tested with both ammonium and nitrite, resulting in four types of tests.

Nitrite formation was studied in two simulated distribution systems (SDSs). Each SDS consisted of a 22-m pipe loop and a covered water tank (material polypropylene). The flow was generated by a magnet pump (IWAKI MD-30RVM-220N, Japan), and measured with a water meter (SUVE SVI 1511, Finland). The SDSs were equipped with pressure gauges (Freescale Semiconductor MXP5050DP, USA) and thermometers (Nokeval Pt100 sensor with a transmitter 620, Finland) (see Fig. 1a). The external diameter of the pipe was 25 mm, the wall thickness was 2.3 mm, and the material was polyethylene (Uponor “blue stripe,” Finland). A vent valve was installed at the highest point of each pipe loop. The SDSs were operated in a closed and temperature–regulated room at the temperature of 18 °C, with a positive water pressure of 0.2 bar and a volumetric flow of 0.1 m^3^ h^-1^. The water was pumped from the bottom of the tank through the pipe loop and returned to the tank below the water level. Five biofilm collectors were installed in the pipe loops, each consisting of 0.6 m of water pipe with PVC valves at both ends. Two similar SDSs, named SDS1 and SDS2, were built in the research laboratory of Water Engineering in Aalto University, Espoo, Finland.

Before starting the operation of the SDSs, the tanks and pipe loops were shock chlorinated and rinsed with tap water, and consequently with water purified with reverse osmosis (RO water). Furthermore, a final rinsing was done by circulating RO water through the SDSs for three weeks to dissolve the remnants of organic carbon and nutrients from the unused materials. To grow the nitrifying biofilm, the SDSs were operated for 30 weeks before nitrification was established. Tap water (30 L) was changed weekly in both SDSs, with ammonium additions to the level of 200 μgN L^-1^. In the SDSs the hydraulic retention time was 18 min and, in the pipe loop, 4 min (the volume of the pipe loop: 7.2 L). The influent water was dechlorinated water. Dechlorination was organized in a temperature-regulated research room by storing the tap water in closed vats for one week before adding it to the SDSs. The vats were rinsed with RO water for several weeks before use. A similar three-day dechlorination method has been used by Zhang et al., (2008). On week 16, phosphorus addition to the level of 5 μgP L^-1^ (Lehtola et al., 2002) was initiated in order to boost the growth of nitrifiers (see the “Supplementary information” section, Table S1). The whole study lasted for 59 weeks.

### 2.2 Nitrification tests

Four types of nitrification test were executed in order to evaluate the nitrite formation (see Fig. 1b). In the tests, two NOM concentrations were applied: the normal NOM concentration of the tap water and a reduced NOM concentration. These were combined with ammonium and nitrite as the initial substrates for nitrification. The NOM in the tap water of the Helsinki Metropolitan Area originates from Lake Päijänne in Southern Finland (coordinates 61°36′50″N, 25°28′55″E). The median concentration of NOM as total non-purgeable organic carbon (TOC) in the freshly produced tap water was 1.6 mg L^-1^ (range: 1.4–2.0 mg L^-1^) during 2010–2015. Furthermore, the nitrogen fractions in the water entering the DWDS during 2010–2015 were: ammonium (including monochloramine) 100–190 μgN L^-1^, nitrite 3–43 μgN L^-1^, nitrate 250–370 μgN L^-1^, and total nitrogen 350–660 μgN L^-1^.

The tests were organized as batch tests, new influent was introduced once a week, and the biofilm inside the pipe loop, once grown, was kept intact. In commencing all tests, the required chemicals were inserted as solutions to the influent water in the tank (see the “Supplementary information” section, Table S1, for the concentrations and volumes of the solutions). Thereafter, the fresh influent was introduced into the biofilm by pumping it through the pipe loop at the maximum flow (1.0 m^3^ h^-1^) for 10 min. Subsequently, the flow was adjusted to 0.1 m^3^ h^-1^ and the first sample taken.

Ammonium tests were started on week 31, with two trial tests. Nitrite tests were initiated on week 35, and after two consecutive nitrite tests, they were performed alternating weeks with ammonium tests (see the timetable in Table S2 in the “Supplementary information” section). In a nitrite test, the water was first introduced into the tank and pumped through the pipe loop with a 0.1 m^3^ h^-1^ flow for 24 h to get rid of the ammonium content of the tap water, utilizing nitrification in the pipe loop.

The NOM concentration of the water was reduced by mixing 15 L of tap water with 15 L of RO water. For example, distilled water has earlier been used for this purpose (Zhang et al., 2010). The RO water was stored for one week in the research room, similarly to the tap water storage. The hardness and alkalinity of the diluted tap water were amended with calcium, magnesium, and hydrogen carbonate ions (see Table S1 in the “Supplementary information” section). In addition, nitrate was amended between weeks 44 and 50.

The SDSs were operated similarly, resulting as two similar nitrification tests each week. This was done particularly because two biological processes tend to drift apart, even though they are operated similarly (Douterelo et al., 2013). Thus, the manipulations were organized sequentially, with a final verification with the initial conditions. Tests with normal NOM were performed first, for eight weeks, and for four weeks in the end. Tests with reduced NOM were performed for eleven weeks in between (see the timetable in the “Supplementary information” section, Table S2).

### 2.3 Water and biofilm sampling and analyses

Nitrite formation was monitored by sampling and analyzing the concentrations of ammonium (NH_4_^+^), nitrite (NO_2_^-^), nitrate (NO_3_^-^), and total nitrogen (N_tot_) from the water in the tanks. For example, Zhang et al., (2008) tracked AOB and NOB activity by measuring the loss of ammonia and the production of nitrite and nitrate. The sampling intervals varied between tests, and lasted 0.75–24 h during the first 1–3 days of the tests, except for the trial tests, which were only sampled with a 24 h interval. The final effluents were sampled when they were removed from the tanks. The nitrogen fractions of water samples were analyzed in the laboratory of Water and Environmental Engineering, according to the methods in Table S3 in the “Supplementary information” section. All the ammonium, nitrite, and nitrate concentrations were reported as nitrogen (see Table 1). The samples were analyzed during the same day, and if the analysis took longer than one day, the samples were stored in 4 °C. Two replicates of each analysis were performed. The concentration of dissolved oxygen and the pH of the water were measured after each sampling. In addition, general water quality was analyzed once a month (for TOC, heterotrophic plate count [HPC], hardness, total residual chlorine [Cl_2_], pH, alkalinity, and turbidity; Table 1). NOM was analyzed as assimilable organic carbon (AOC) and TOC from tap water and the influent during the tests with reduced NOM in a sampling campaign (weeks 47–50). All the water analysis methods are related in the “Supplementary information” section, Table S3. After all of the tests, in week 59 the biofilm collectors were sampled according to the description in the “Supplementary information” section (Section 3). The microbial community of biofilms were analyzed for the share of AOB, NOB, and the most abundant bacterial species, according to the description in Table S4 in the “Supplementary information” section. Other analyses performed from the biofilm samples were a total bacterial count with the DAPI staining and suspended solids (SS) (see Table S4 in the “Supplementary information” section).

**Table 1:** The influent water quality in the nitrification tests with normal and reduced NOM (md = median).

Water quality analyses											
Tests with normal NOM											
	Turbidity	Alkalinity		pH		Total Cl_2_	Hardness		TOC		HPC
	FNU	mmol L^-1^				mg L^-1^	mmol L^-1^		mg L^-1^		cfu mL^-1^
md	0.21	0.70		7.8		0.02	0.51		1.6		6900
min	0.16	0.45		7.6		0.02	0.41		1.5		1000
max	0.27	0.76		8.0		0.03	0.53		2.1		27 000
N	6	6		6		6	6		6		8
Tests with reduced NOM											
md	0.15	0.71		8.0		0.02	0.50		1.0		9500
min	0.11	0.69		7.9		0.02	0.46		0.9		4500
max	0.27	0.71		8.0		0.02	0.54		1.1		18 000
N	6	6		6		6	6		12		8
Nitrogen analyses											
Ammonium tests						Nitrite tests					
Tests with normal NOM											
	NH_4_^+^	NO_2_^-^	NO_3_^-^		N_tot_		NH_4_^+^	NO_2_^-^		NO_3_^-^	N_tot_
	μgN L^-1^	μgN L^-1^	μgN L^-1^		μgN L^-1^		μgN L^-1^	μgN L^-1^		μgN L^-1^	μgN L^-1^
md	210	40	390		760		<5	77		470	660
min	180	12	310		710		<5	70		450	630
max	300	70	450		810		<5	110		510	710
N	14	14	14		14		10	10		10	10
Tests with reduced NOM											
md	200	33	320		630		<5	75		360	520
min	160	20	200		500		<5	71		230	370
max	220	72	390		650		<5	80		390	540
N	12	12	12		12		10	10		10	10

### 2.4 Calculations and statistical methods

Apparent reaction rates were calculated by fitting a line in the first linear part of the nitrite concentration changes in the ammonium tests. The slope of the line was taken as the rate:

(3) *r* = *m*_NO2-_

where *r* = apparent reaction rate (μgN L^-1^h^-1^) and *m*_NO2-_ = the slope of the nitrite concentration change (μgN L^-1^h^-1^). The apparent rates were calculated for four or five pairs of time and concentration.

In addition, nitritation and nitratation reactions were inspected with a model of two consecutive and irreversible first order reactions (Equations (4), (5), (6)). Pseudo-first order modeling is often used, for example, when the biodegradation of micropollutants is examined (Ternes and Joss, 2006). For simplicity, we approximated the SDSs as completely mixed reactors and the concentrations in the tank were taken as the concentrations of the effluent. This assumption caused a small error, however, since all the tests were treated equally, they are comparable to each other. The pseudo-first order kinetic equation for nitritation follows:

(4) [NH_4_^+^]_t_ = [NH_4_^+^]_0_ exp(-*k*_NH4+_*t*),

where [NH_4_^+^]_t_ = ammonium concentration at time *t* (μgN L^-1^), [NH_4_^+^]_0_ = the initial ammonium concentration (μgN L^-1^), *k*_NH4+_ = the pseudo-first order reaction rate constant for ammonium (h^-1^), and *t* = time (h). The pseudo-first order kinetic equation for two consecutive reactions, nitritation and nitratation, follows:

[NO_2_^-^]_t_ = [NO_2_^-^]_0_ exp(*k*_NO2-_*t*) + [NH_4_^+^]_0_ (*k*_NH4+_/(*k*_NO2-_ - *k*_NH4+_) (exp(-*k*_NH4+_*t*) - exp(*k*_NO2-_*t*)),

(5) *k*_NH4+_ ≠ *k*_NO2-_,

where [NO_2_^-^]_t_ = nitrite concentration at time *t* (μgN L^-1^), [NO_2_^-^]_0_ = the initial nitrite concentration (μgN L^-1^), and *k*_NO2-_ = pseudo-first order reaction rate constant for nitrite (h^-1^). The pseudo-first order kinetic equation for nitrate formation was calculated with the nitrogen balance as follows:

(6) [NO_3_^-^]_t_ = [NH_4_^+^]_0_ + [NO_2_^-^]_0_ + [NO_3_^-^]_0_ - [NH_4_^+^]_t_ - [NO_2_^-^]_t_,

where [NO_3_^-^]_t_ = nitrate concentration at time *t* (μgN L^-1^), [NO_3_^-^]_0_ = initial nitrate concentration (μgN L^-1^). The theoretical maximum nitrite concentration occurs according to the two consecutive pseudo-first order reactions model at the time *t*_max_, with a zero initial nitrite concentration as follows:

(7) *t*_max_ = 1/(*k*_NH4+_ - *k*_NO2-_) ln(*k*_NH4+_ / *k*_NO2-_).

In the tests with nitrite as the initial substrate, the pseudo-first order reaction rate constants for nitrite, *k*’_NO2-_, were calculated from Equation (8), as follows:

(8) [NO_2_^-^]_t_ = [NO_2_^-^]_0_ exp(-*k*’_NO2-_*t*).

Equations (4) and (8) were solved for *k*_NH4+_ or *k*’_NO2-_ in the logarithmic form. Equation (5) was solved for *k*_NO2-_ utilizing the least squares method. The theoretical maximum nitrite concentrations for each pair of *k*_NH4+_ and *k*_NO2-_ were calculated by fitting *t*_max_ from Equation (7) into Equation (5) as *t*.

The error of the nitrogen balance during the whole test period was calculated to evaluate the possibility of reactions that increase or decrease nitrogen in the water phase, according to Equation (9), as follows:

(9) *ε* = N(influent) -N(samples) – N(effluent),

*ε* is the nitrogen balance error (μg), N(influents) is the sum of nitrogen of all the influent waters (μg), N(samples) is the sum of nitrogen taken in all of the water samples (μg), N(effluent) is the sum of nitrogen of all the removed effluent waters (μg). The sum of nitrogen was calculated by weighing the individual nitrogen concentrations by the corresponding water volume.

The data sets of reaction rates and reaction rate constants were tested for normality with the Anderson–Darling test. The normally distributed data (the pseudo-first order reaction rate constants of nitrite, with normal and reduced NOM and with ammonium and nitrite as the initial substrate, and the separated data of SDS1 and SDS2 with normal and reduced NOM) were compared with Student’s t-test and non-normally distributed (the apparent reaction rates of nitrite and the pseudo-first order reaction rate constants of ammonium with normal and reduced NOM) with Wilcoxon’s rank-sum test. Data was collected in Microsoft Excel, testing and data analysis were performed in MathWorks Matlab (version R2019a), and the figures were finalized in Adobe Illustrator CC.

## 3 Results and discussion

### 3.1 Nitrite formation

**Fig. 2:**
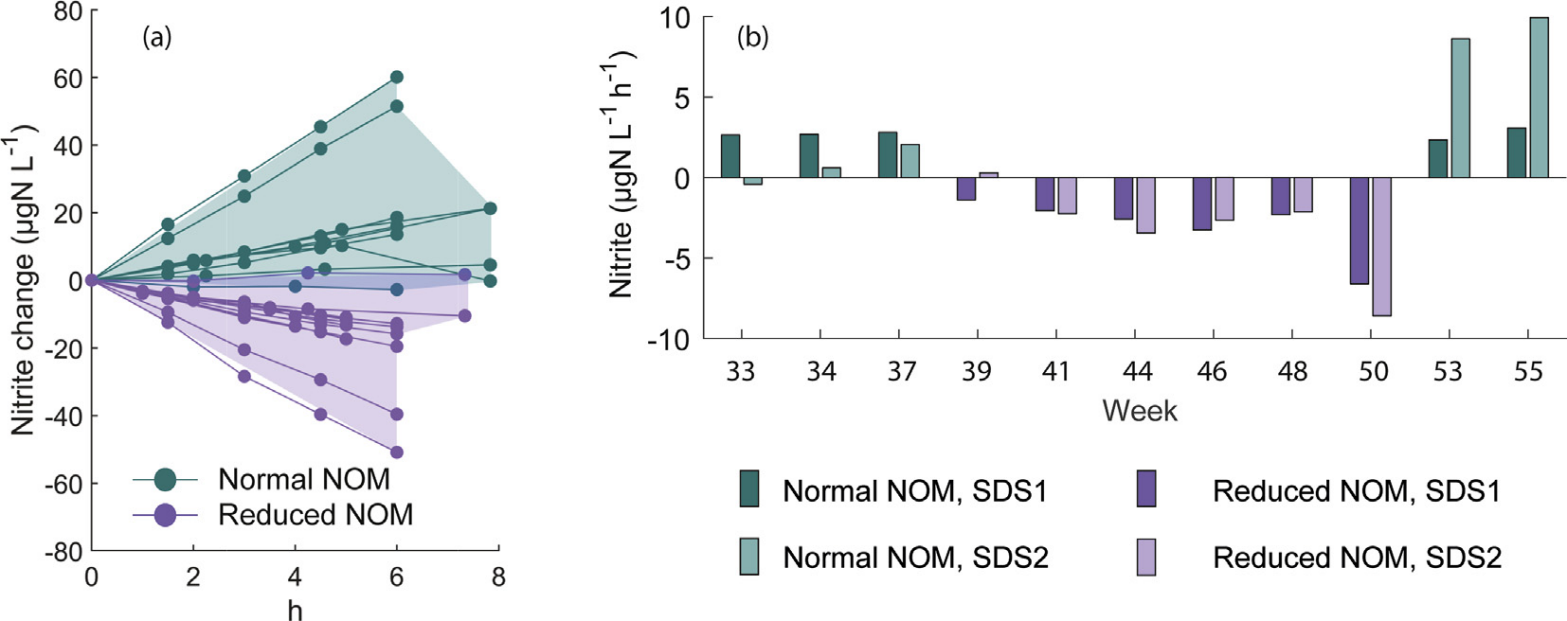
The nitrite formation in the nitrification tests with ammonium as the nitrogen source: (a) nitrite concentration change from the initial value (the ranges of concentration changes with normal and reduced NOM are highlighted with shaded areas), and (b) the slope of the nitrite change in the chronological order of the nitrification tests.

Nitrite formation responded consistently to the changes in NOM concentrations in the nitrification tests with ammonium. With the normal NOM of the tap water (median NOM: 1.6 mg L^-1^, as TOC) nitrite increased, while with reduced NOM (median NOM: 1.0 mg L^-1^, as TOC) nitrite decreased (see Fig. 2a). The nitrite concentrations increased with normal NOM by 2.7 μgN L^-1^ h^-1^, and decreased with reduced NOM by -2.4 μgN L^-1^ h^-1^ as medians (see Fig. 2b). The difference of the concentration changes was statistically significant (Wilcoxon’s rank-sum test: *zval* = -3.4, *p* = 6.5 × 10^-4^). The apparent nitrite formation rates were evaluated for the initial linear part of the concentration profile as the slope of the linear regression line (see Equation (3)).

It is conceivable that the biofilm composition in the pipe loop reacted quickly to the reduced or increased NOM because the heterotrophic bacteria have a significantly higher maximum growth rate than the AOB and NOB: the maximum growth rate of the AOB and NOB is 0.2–0.9 g new cells (g cells d)^-1^ and for the heterotrophic bacteria it is 3.0–13.2 g new cells (g cells d)^-1^ (Tchobanoglous et al., 2003). Nevertheless, the two SDSs had slightly different patterns of nitrite build up or loss even though they were operated similarly. SDS2 produced a wider range of rates (normal NOM: -0.4 to +9.9 μgN L^-1^ h^-1^; reduced NOM: -8.6 to +0.3 μgN L^-1^ h^-1^) than SDS1 (normal NOM: +2.3 to +3.1 μgN L^-1^ h^-1^; reduced NOM: -6.6 to -1.3 μgN L^-1^ h^-1^). SDS2 demonstrated initially slower rates than SDS1, also when the NOM concentrations were first reduced. On the other hand, in the last three tests, the rates were slower in SDS1 than in SDS2. However, in its entirety, the apparent nitrite formation rates did not differ significantly between SDS1 and SDS2 (normal NOM: Student’s *t* = -0.68, *p* = 0.53; reduced NOM: Student’s *t* = 0.19, *p* = 0.85). The ranges of the apparent rates in SDS1 and SDS2 demonstrated that the test design was appropriate. It would possibly have been difficult to compare two SDSs with different water qualities when the two SDSs with the same water quality differed as observed.

### 3.2 Comparing the ammonium and nitrite oxidation reactions

#### 3.2.1 The ammonium tests

**Fig. 3:**
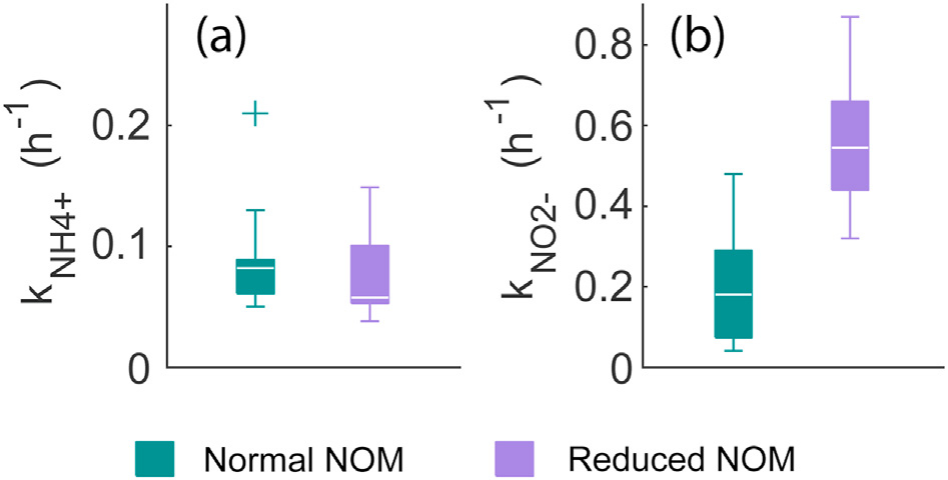
The figures depict (a) the pseudo-first order ammonium oxidation rate constants in the ammonium tests as box and whiskers plots (*N* = 14 with normal NOM; *N* = 12 with reduced NOM) and (b) the pseudo-first order nitrite oxidation rate constants in the ammonium tests (*N* = 14 with normal NOM; *N* = 12 with reduced NOM).

When the ammonium tests were interpreted with the pseudo-first order reaction rate models (Equations (4), (5), (6)), it was found out that the decrease of nitrite concentrations with reduced NOM was a result of nitrite being oxidized more rapidly. The values of the reaction rate constant of nitrite (*k*_NO2-_, median with normal NOM: 0.18 h^-1^ and with reduced NOM: 0.55 h^-1^) differed significantly from each other (see Fig. 3b; Student’s *t* = -6.2, *p* = 2.2 × 10^-6^). On the other hand, the values of the reaction rate constant of ammonium (*k*_NH4+_) with normal NOM (median 0.082 h^-1^) and reduced NOM (median 0.058 h^-1^) did not differ significantly (see Fig. 3a; Student’s *t* = 0.9, *p* = 0.37). This indicated that the differences of the nitrite concentrations in the nitrification tests were mainly caused by changes in the nitrite oxidation rates and not by changes in ammonium oxidation. All the model fittings can be seen in Fig. S1 and S2 in the “Supplementary information” section. The trial tests with ammonium were also included in the pseudo-first order modeling.

Interestingly, the ammonium oxidation was not affected significantly by the decreased NOM but only nitrite oxidation. One probable factor behind this observation is the free energy of transformation (ΔG⁰’), which is -290.4 kJ mol^-1^ for ammonium oxidation, and only -72.1 kJ mol^-1^ for nitrite oxidation (Stumm and Morgan, 2009). Thus, ammonium oxidation is thermodynamically four times more favorable than nitrite oxidation. It is possible that the NOB were more sensitive to the changes in their environment because they gained less energy than the AOB. Furthermore, the NOB have been observed to be impacted by a lower monochloramine concentration than the AOB at 25 °C (Gomez-Alvarez et al., 2014; Schrantz et al., 2013). This also indicates that the NOB may be generally more vulnerable to changes in the environment.

#### 3.2.2 The nitrite tests

**Fig. 4:**
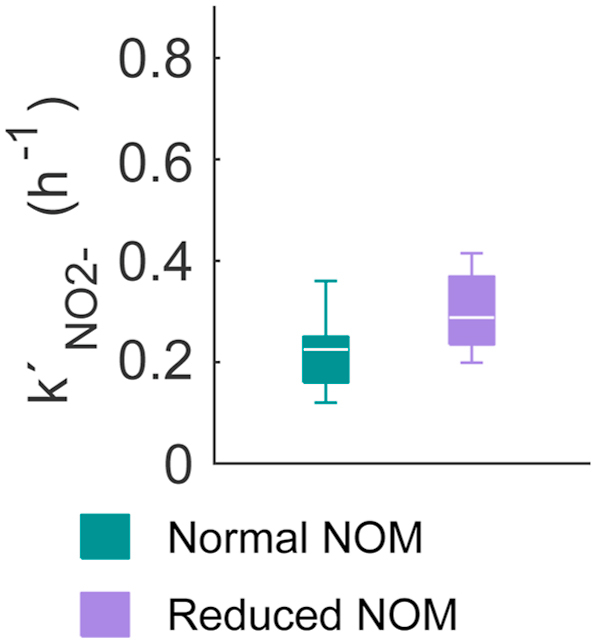
The pseudo-first order nitrite oxidation rate constants in nitrite tests, as a box and whiskers plot. The comparison of the values of *k*’_NO2-_, acquired in the nitrite tests with the normal and reduced NOM (*N* = 10 with normal NOM; *N* = 10 with reduced NOM).

The nitrification tests with nitrite as the initial substrate were interpreted with the pseudo-first order reaction rate model (Equation (8)). The nitrite tests confirmed that there was a significant difference in the values of the reaction rate constants (*k*’_NO2-_) with normal and reduced NOM (Student’s *t* = -2.1, *p* = 0.050; see Fig. 4). This finding supported the observations from the ammonium tests. All the model fittings can be seen in Figs. S3 and S4 in the “Supplementary information” section.

In the nitrite tests, the median of *k*’_NO2-_ was 0.23 h^-1^ with normal NOM, and 0.29 h^-1^ with reduced NOM (see Fig. 4). Thus, the means of *k*_NO2-_ and *k*’_NO2-_ differed by -0.02 h^-1^ with normal NOM and 0.25 h^-1^ with reduced NOM. The origin of nitrite may partly explain the difference of the *k*_NO2-_ and *k*’_NO2-_ with reduced NOM. In the nitrite tests, the nitrite originated entirely from the water phase, contrary to the ammonium tests where most of the nitrite was created inside the biofilm. Thus, nitrite was more readily available for the NOB in the ammonium tests because the rates lacked the material transfer resistance of nitrite from the water phase to the insides of the biofilm. Apparently, this explanation is not necessarily relevant for normal NOM. However, it is evident that *k*_NO2-_ and *k*’_NO2-_ cannot be used interchangeably.

### 3.3 Estimating the theoretical maximum nitrite concentrations

The maximum concentrations of nitrite are of interest, because they are a potential health hazard in distributed water. The conditions leading to high maximum concentrations are to be avoided if possible.

**Fig. 5:**
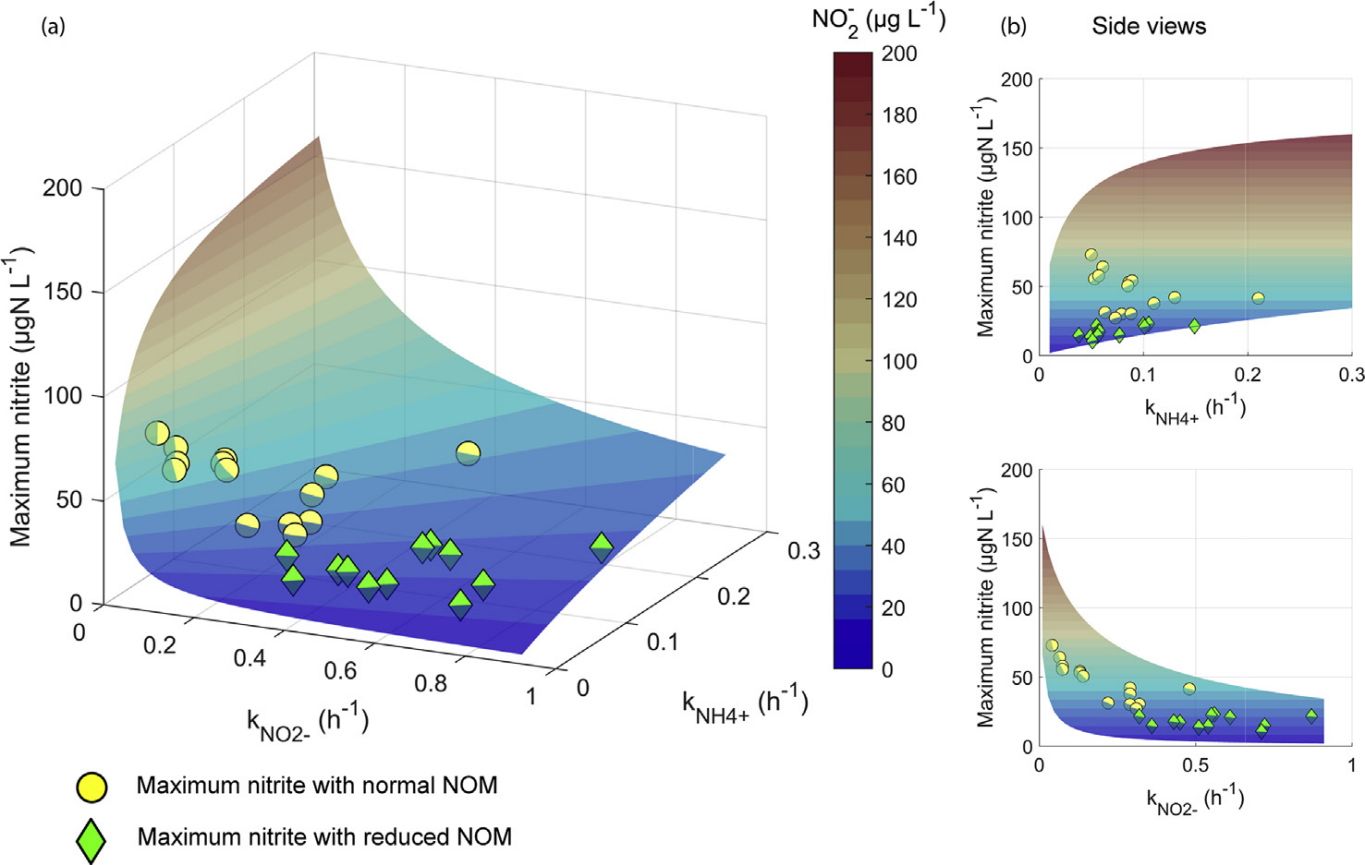
The figures depict (a) the theoretical maximum nitrite concentration (the z-axis) with *k*_NH4+_ and *k*_NO2-_ shown as a 3D surface. The reaction rate constants (*k*_NH4+_ and *k*_NO2-_) calculated from the ammonium tests are indicated by circles and diamonds. The circles and the diamonds are on the surface; the bright part is above the surface, and the shaded part is behind the surface. Side views (b) and (c) are included to help in interpreting the 3D image. The 3D image is elucidated in more detail in the “Supplementary information” section (in Figs. S5 and S6).

The pseudo-first order model, described above, allowed us to estimate a theoretical maximum of the nitrite concentration (Equations (5) and (7)) by varying *k*_NH4+_ and *k*_NO2-_. The model was applied with the initial ammonium concentration of 180 μgN L^-1^ with no nitrite (see Fig. 5). The maximum nitrite concentrations formed a curved surface on which the lowest maximum nitrites occurred when *k*_NH4+_ was low and *k*_NO2-_ was high; on the other hand, the highest concentration was with a combination of high *k*_NH4+_ and low *k*_NO2-_.

The nitrite concentrations are formed as an intermediate reaction product, and thus they can form a peak when the initial nitrogen compound is ammonium. If the nitrite oxidation rate is high enough compared with the ammonium oxidation rate, the peak may be non-existent and no extra nitrite is formed. The ratio of the reaction rate constants of the two consecutive reactions (*k*_NH4+_:*k*_NO2-_) determined whether nitrite concentration started to increase or decrease initially. Theoretically, in the case of the first trial test with ammonium in SDS1 (week 31; Fig. S1a in the “Supplementary information” section), if the ratio *k*_NH4+_:*k*_NO2-_ had been 0.06 or less, nitrite would have initially started to decrease. Here it was 0.94, thus producing a peak in the nitrite concentration profile. Altogether, the median of the ratios of *k*_NH4+_:*k*_NO2-_ was 0.53 in the tests with normal NOM and 0.13 with reduced NOM.

### 3.4 The biofilms

In the biofilm samples—taken after the experiments, on week 59, and combined from the biofilms of SDS1 and SDS2—the relative abundance of the AOB (*Nitrosomonas* sp.) was 9.3%, and the relative abundance of NOB (*Nitrospira* sp.) was 8.7%. The high shares of AOB and NOB demonstrated the existence of nitrification activity in the biofilm. The most abundant species was the heterotrophic bacteria *Bradyrhizobium* sp*.* (25%), which has earlier been found in abundance in non-disinfected SDSs (Aggarwal et al., 2018; Gomez-Alvarez et al., 2014). However, in a full-scale study encompassing 18 months and 5 samples, this species was not found (Kelly et al., 2014), probably because monochloramine was used for secondary disinfection. The rest of the biofilm microbiological composition in our tests is related in the “Supplementary information” section (Section 5).

The total numbers of microbial cells (with DAPI staining) in the biofilms were 5.9 × 10^8^ cells m^-2^ in SDS1 and 6.8 × 10^8^ cells m^-2^ in SDS2. Furthermore, the total solids of the biofilms were 180 mg m^-2^ in SDS1 and 240 mg m^-2^ in SDS2. Combined with the share of AOB and NOB above, we can estimate the number of *Nitrosomonas* sp. (3.1 × 10^8^ cells g^-1^ in SDS1 and 2.6 × 10^8^ cells g^-1^ in SDS2) and *Nitrospira* sp. (2.9 × 10^8^ cells g^-1^ in SDS1 and 2.5 × 10^8^ cells g^-1^ in SDS2). For example, Lipponen et al. (2002) enumerated 4.2 × 10^4^–2.2 × 10^7^ MPN g^-1^ of AOB, and 1.8 × 10^3^–2.3 × 10^8^ MPN g^-1^ of NOB in the sediment samples of four non-chlorinated DWDSs. Our results are analogous with these results, taking into account that the latter probably underestimate the share of NOB and AOB in the biofilm by including inorganic sediments in the mass of the sample.

The analyses of the biofilm did not, however, represent the whole test period. Deducing from the varying nitrite formation rates, the biofilms inside the pipe loops went through changes during the tests. There were no changes in the way the pipe loops were operated during each test period, thus, the change in the nitrite formation rates was most probably due to the biofilm change. Nevertheless, the variation in nitrite formation originating from the biofilm changes was included in the statistical variation of all the replicates of each type of nitrification test.

In full-scale DWDSs, the microbial composition of the biofilm varies along the lengthy pipes with the changing concentrations of the distributed water (Douterelo et al., 2016; Gomez-Alvarez et al., 2014). In the SDSs of the current study, the biofilm was likely more uniform throughout the length of the pipe loops, though variation with time occurred, as mentioned above. The nitrite profiles with normal NOM were relatively similar to nitrite formation profiles observed in a monochloraminated full-scale DWDS (Rantanen et al., 2018). Nitrite peaked at the water age of 5.0–6.7 h at full scale, while in the current study the nitrite peak occurred at 4–14 h. The waters in the tests and at full scale both originated from the same water treatment plant (WTP), with the difference of monochloramine being used at full scale.

The concentration of AOB and NOB in the water may have had an effect on the ammonia and nitrite oxidation rates. However, we considered this effect minor. Before the actual tests, the biofilm was grown in the pipes for a six-month period, during which ammonium reduction or nitrite formation were not observed. Thus, notable nitrification was not achieved in the water phase by the suspended AOB and NOB.

One possible explanation for decreased nitrite concentrations with reduced NOM could be that nitrite ions were more available for the NOB. If the heterotrophic matrix of the biofilm was less dense, the AOB would have generally been closer to the NOB. Thus, the material transfer of nitrite ions from AOB to NOB would have generally been faster. A related effect has been observed in nitrifying activated sludge plants when the supply of nitrite to NOB deteriorated during a process disturbance (Kaelin et al., 2009; Knapp and Graham, 2007).

### 3.5 The applicability of the models

The apparent and pseudo-first rate models allowed us to interpret the changes that the reduced NOM had on the biofilm, resulting in differences in nitrite formation. The other conditions (temperature, flow, total residual chlorine, alkalinity, and hardness) were controlled in order to only observe the consequences of reduced NOM. The applicability of the pseudo-first rate models was evaluated with ammonium test data on the first eight tests (weeks 31–34) with one or more samples after the first day (see Figs. S1 and S2 in the “Supplementary information” section). The coefficients of determination (*R*^2^) for nitrite formation in tests with ammonium ranged from 0.57 to 0.99 (median: 0.90; *N* = 8). Thus, it was considered that the pseudo-first rate model was sufficient to describe the data of the tests that lasted one week.

Usually, the growth rate of the microbes is included in modeling nitrification in DWDSs (Schrantz et al., 2013; Wahman et al., 2011; Woolschlager et al., 2001; Yang et al., 2008a). This is mainly needed for long-term prediction. However, in our research we wanted to compare two conditions, and using simpler reaction rate models was sufficient for this. Also, the pseudo-first order model was the simplest possible model that could separate the ammonium and nitrite reaction rates from each other.

### 3.6 The natural organic matter

The NOM concentration in tap water and influent water were analyzed in a sampling campaign during the tests with reduced NOM as TOC and AOC (weeks 47–50). The target was to compare the tap water and diluted influent from exactly the same tap water. The TOC concentration was reduced from 1.9 mg L^-1^ to 1.1 mg L^-1^, while AOC was reduced from 150 μgC L^-1^ to 120 μgC L^-1^ as medians. This confirmed that the experimental procedure was able to reduce the NOM content of the influent. AOC is supposed to be the main source for biofilm regrowth by heterotrophic bacteria (Ross et al., 2013). According to the results of the sampling campaign, the difference in AOC was not as remarkable as the difference in TOC. The minor difference in AOC may have been a result of the relatively high phosphorus content of the sample waters (5 μg L^-1^) compared with ordinary drinking water in Finland (below the LoQ of 0.5 μg L^-1^). It has been reported that normally phosphorus is the limiting nutrient in AOC assays, not NOM (Miettinen et al., 1996). Nevertheless, the NOM dilution method of the current study did not alter the general size distribution of the NOM molecules, thus the observations were not attributable to changes in the molecular size range, as Huang et al. (2016) had noticed.

### 3.7 The possibility of denitrification and other reactions of nitrogen

The nitrogen balances were calculated to evaluate the possibility of reactions that are capable of changing the total amount of nitrogen in the water phase (denitrification, anammox, and nitrogen accumulation). As detected, the biofilm contained bacteria capable of these reactions (see the “Supplementary information” section, Section 5). The total balance error of nitrogen inputs and outputs during the whole tests was calculated according to Equation (9). A minor amount of nitrogen was increased (total nitrogen in SDS1: +1.1% of the feed; total nitrogen in SDS2: +2.0% of the feed; inorganic nitrogen in SDS1: +0.3% of the feed; inorganic nitrogen in SDS2: +1.0% of the feed). The total amounts of increase were, however, small compared to the weekly variation of the balances (total nitrogen in SDS1: -5.6% to +8.9% of the feed; total nitrogen in SDS2: -5.0% to +7.2% of the feed; inorganic nitrogen in SDS1: -5.0% to +4.6% of the feed; inorganic nitrogen in SDS2: -7.0% to +8.1% of the feed). Thus, the effect of the reactions accumulating or releasing nitrogen were not significant as a whole.

Although denitrifying organisms and denitrification have been observed in DWDSs (Nagymate et al., 2016) and SDSs (Masters et al., 2015), the reactions requiring low oxygen concentrations (the denitrification of nitrite or nitrate and anammox) were not likely because the minimum oxygen concentration of the water was 8.8 mgO_2_ L^-1^. Thus, the anoxic conditions were only possible in the deep parts of the biofilm, and these are strongly limited by mass transfer into and out of the biofilm.

Because nitrite formed in the tests, a one-step complete ammonium oxidation (comammox) reaction (Daims et al., 2015) was unlikely with normal NOM. However, the occurrence of comammox could not be excluded in the tests with reduced NOM, especially because *Nitrospira* sp., the type of bacteria capable of comammox, was observed in the biofilm.

## 4 Conclusions

The effects of natural organic matter (NOM) on nitrite formation in the conditions of non-disinfected tap water were studied in simulated distribution systems (SDSs), with an emphasis on both ammonium and nitrite oxidation. The tests revealed that reducing the NOM concentration declined the nitrite levels. Furthermore, this research suggested that nitrite oxidation dominated nitrite formation in the conditions of water distribution.

The results of this research emphasize the benefits of NOM removal at water treatment plants (WTPs). Decreasing NOM at WTPs with non-disinfected drinking water distribution systems (DWDSs) could constitute a promising alternative for reducing nitrite concentrations in the distributed water. Furthermore, the observed strong dependence of nitrite concentrations on nitrite oxidation is a fruitful starting point for analyzing nitrification episodes in disinfected DWDSs.

## Declaration of competing interest

The authors declare that they have no known competing financial interests or personal relationships that could have appeared to influence the work reported in this paper.
