# Supplementary material for: Decreased natural organic matter in water distribution decreases nitrite formation in non-disinfected conditions, via enhanced nitrite oxidation
Source: Water Res X. 2020 Sep 30;9:100069. doi: 10.1016/j.wroa.2020.100069 (PMC7552091; doi:10.1016/j.wroa.2020.100069)
Supplement: Multimedia component 1 [file mmc1.pdf]

Decreased natural organic matter in water distribution decreases nitrite formation in non-disinfected conditions, via enhanced nitrite oxidation

Pirjo-Liisa Rantanen[a], Minna M. Keinänen-Toivola[b], Merja Ahonen[b], Alejandro Gonzalez-Martinez[c], Ilkka Mellin[d], Riku Vahala[a]

[a] Department of Built Environment, Aalto University, PO Box 15200, FI-00076 Aalto, Finland

[b] Faculty of Technology, Satakunta University of Applied Sciences, PO Box 1001, FI-28101 Pori, Finland

[c] Department of Microbiology, University of Granada, Campus Universitario de Cartuja, 18071 Granada, Spain

[d] Department of Mathematics and Systems Analysis, Aalto University, PO Box 11100, FI-00076 Aalto, Finland

Corresponding email: pirjo.rantanen@aalto.fi

Supplementary information

Section 1: Additions to the influent water and the timetable

Table S1. Additions in the ammonium and nitrite tests. (a1) = ammonium test with Normal NOM, (a2) = ammonium test with Reduced NOM, (n1) = nitrite test with Normal NOM, (n2) = nitrite test with Reduced NOM. * weeks 31 – 43, ** weeks 44 – 56, *** weeks 44 – 50.

Test / chemical	NH_4-N	NO_2-N	Ca^{2+}	Mg^{2+}	HCO_3^-	P	NO_3-N
**Concentration**	4.56 mg L^{-1} NH_4Cl	10.93 mg L^{-1} KNO_2	35.9 g L^{-1} $CaCl_2x2H_2O$	7.60 g L^{-1} $MgSO_4x7H_2O$	3.17 g L^{-1} $NaHCO_3$	(1) 4.39 g L^{-1} KH_2PO_4<br>(2) 1.34 g L^{-1} $NaH_2PO_4xH_2O$	5.59 mg l^{-1} $NaNO_3$
**(a1)**	5 ml					150 µl (1) *<br>0.5 ml (2) **	
**(a2)**	5 ml		30 ml	30 ml	300 ml	150 µl (1) *<br>0.5 ml (2) **	5 ml ***
**(n1)**		5 ml				150 µl (1) *<br>0.5 ml (2) **	
**(n2)**		5 ml	30 ml	30 ml	300 ml	150 µl (1) *<br>0.5 ml (2) **	5 ml ***

Table S2. The timetable of the ammonium and nitrite tests with normal and reduced NOM.

	Ammonium tests				Nitrite tests			
	Normal NOM		Reduced NOM		Normal NOM		Reduced NOM	
	N	Test week	N	Test week	N	Test week	N	Test week
Trial tests	4	31						
		32						
Tests	10	33	12	39	10	35	10	40
		34		41		36		43
		37		44		38		45
		53		46		54		47
		55		48		56		49
				50				

Section 2: Water analyses

Table S3. Analytical methods utilized in water analyses.

Analysis	Method	Device/method	Place
Total nitrogen (N_{tot})	SFS-EN ISO 11905-1, 1998 and Standard Methods (2005) 4500 B. Ultraviolet Spectrofotometric Screening Method	Lange Ganimede N	Water engineering, Aalto University, Espoo, Finland
Nitrite nitrogen (NO_2^--N)	SFS-EN ISO 13395, 1997	Flow injection analysis method with FOSS Tecator, FIAstar 5000 Analyzer and Sampler 5027	-"-
Nitrite + nitrate nitrogen (NO_2^- + NO_3^- -N)	SFS-EN ISO 13395, 1997	Flow injection analysis method with FOSS Tecator, FIAstar 5000 Analyzer and Sampler 5027	-"-
Ammonium nitrogen (NH_4^+-N)	ISO 7150/1, 1984	Shimadzu UV-1201 UV-VIS-spectrophotometer	-"-
HPC	SFS-EN ISO 6222, 1999	R2A growth medium, pour-plate method	-"-
Alkalinity	SFS-EN ISO 9963-1, 1996	pH meter WTW inoLab pH 720, probe Sentix 81 Plus, burette Metrohm Dosimat 775	-"-
Hardness	SFS 3003, 1987	Burette Metrohm Dosimat 775	-"-
UV adsorption	SFS-EN ISO 7027, 2000	Hach 2100AN IS Turbidimeter	-"-
Total chlorine (Cl_2)	SFS-EN-ISO 7393-2, 2000	Shimadzu UV-1201 UV-VIS-spectrophotometer	-"-
Total non-purgeable organic carbon (TOC)	SFS-EN 1484, 1997	Total organic carbon analyzer Shimadzu TOC-VCPH + ASI-V	-"-
pH	SFS-EN ISO 9963-1, 1996	pH meter WTW inoLab pH 720, probe Sentix 81 Plus	-"-
Turbidity	SFS-EN ISO 7027, 2000	Hach 2100AN IS Turbidimeter	-"-
Total phosphorus (P_{tot})	SFS-EN ISO 6878, 2004	Shimadzu UV-1201 UV-VIS-spectrophotometer	-"-
Assimilable organic carbon (AOC)	(Van Der Kooij, et al., 1982; Miettinen, et al., 1999)		National Institute for Health and Welfare (THL), Kuopio, Finland

Section 3: Biofilm sampling and analyses

The biofilm collectors were sampled once, as the final step of the study, for both of the SDSs. The collectors were disconnected, and closed with the valves on both of the ends of the collectors. The water contents of the collectors were kept intact, if possible. The collectors were packed carefully and sent via a courier to National Institute for Health and Welfare (THL) in Kuopio. The extraction of the biofilm samples was started on the following day.

At THL, the biofilm samples were extracted from the biofilm collector pipes by shaking with 2-mm, sterile glass beads in three positions for 10 min each position (plane shaker Edmund Bühler, 250 rpm), recovering the fluid and finally rinsing with 5 ml of sterile ion-free water, which was added to the recovered fluid. The filtered samples were frozen to -20°C and transported to the Water laboratory of Aalto University, and stored in -20°C until analyzed.

Table S4. The methods of the biofilm analyses.

**Analysis**	**Method**	**Device**	**Place**
Total cell count (DAPI)	(Hobbie, et al., 1977)		National Institute for Health and Welfare (THL), Kuopio, Finland
qPCR	DNA extraction and sequencing with the method in Kruglova et al. (2017)		Research & Testing Laboratory, Lubbock, TX, USA
Suspended solids (SS)	SFS-EN 872, 2005	GF/C glass fiber filter	Water engineering, Aalto University, Espoo, Finland

Section 4: Analyzed data and the pseudo-first order models

All the analyzed nitrite concentrations eventually decreased below the LoQ. The final samples, taken a week after the first day, are not shown in the Figures S1-S4, to show the first data points with clarity. Data files are provided at http://dx.doi.org/10.5281/zenodo.3894530.

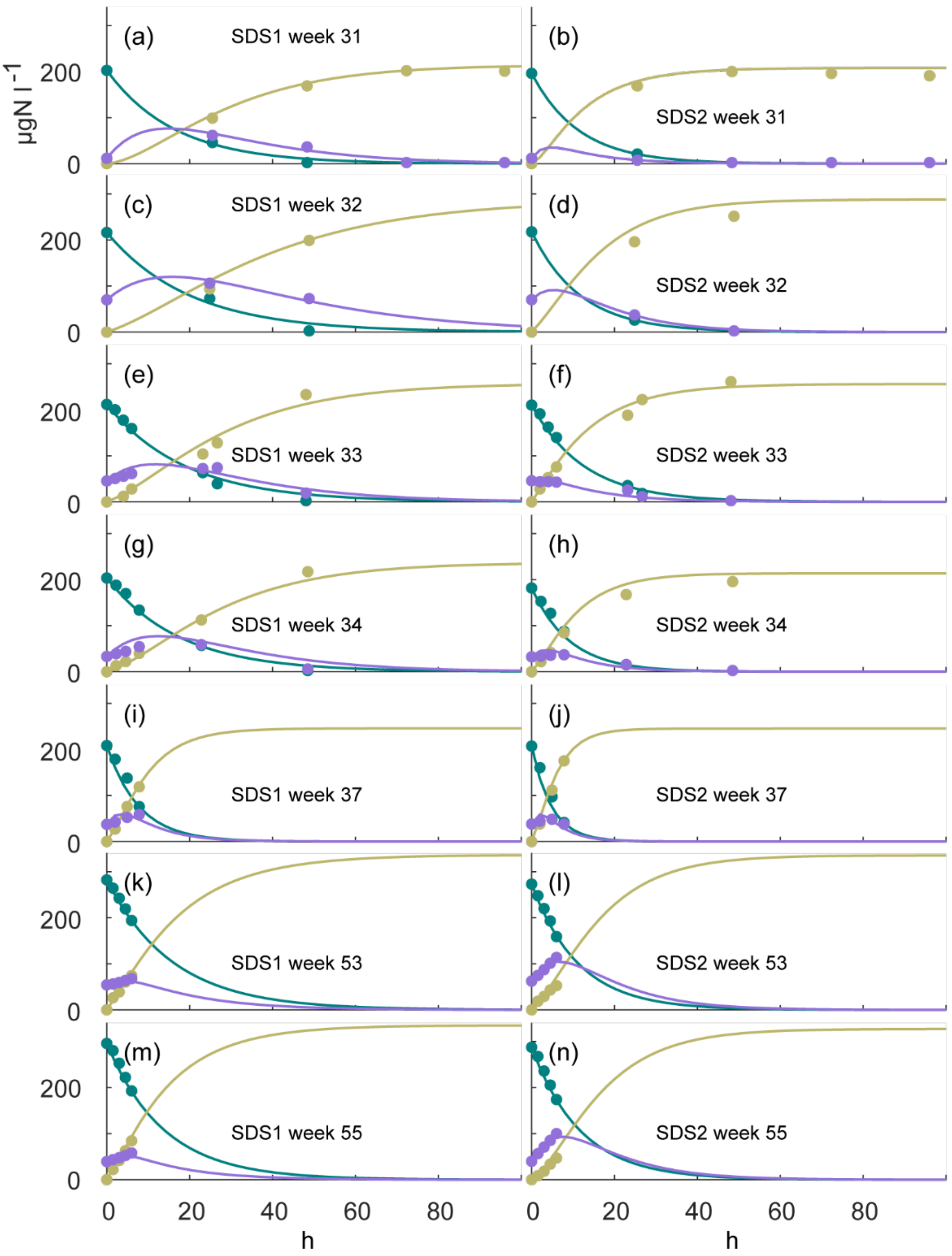

Figure S1. The data of the ammonium tests and the fitted pseudo-first order reaction rate models of the ammonium, nitrite and nitrate concentrations, with Normal NOM. Teal dots: measured ammonium, teal line: modeled ammonium, purple dots: measured nitrite, purple line: modeled nitrite, dark yellow dots: measured nitrite increase, and dark yellow line: modeled nitrate increase.

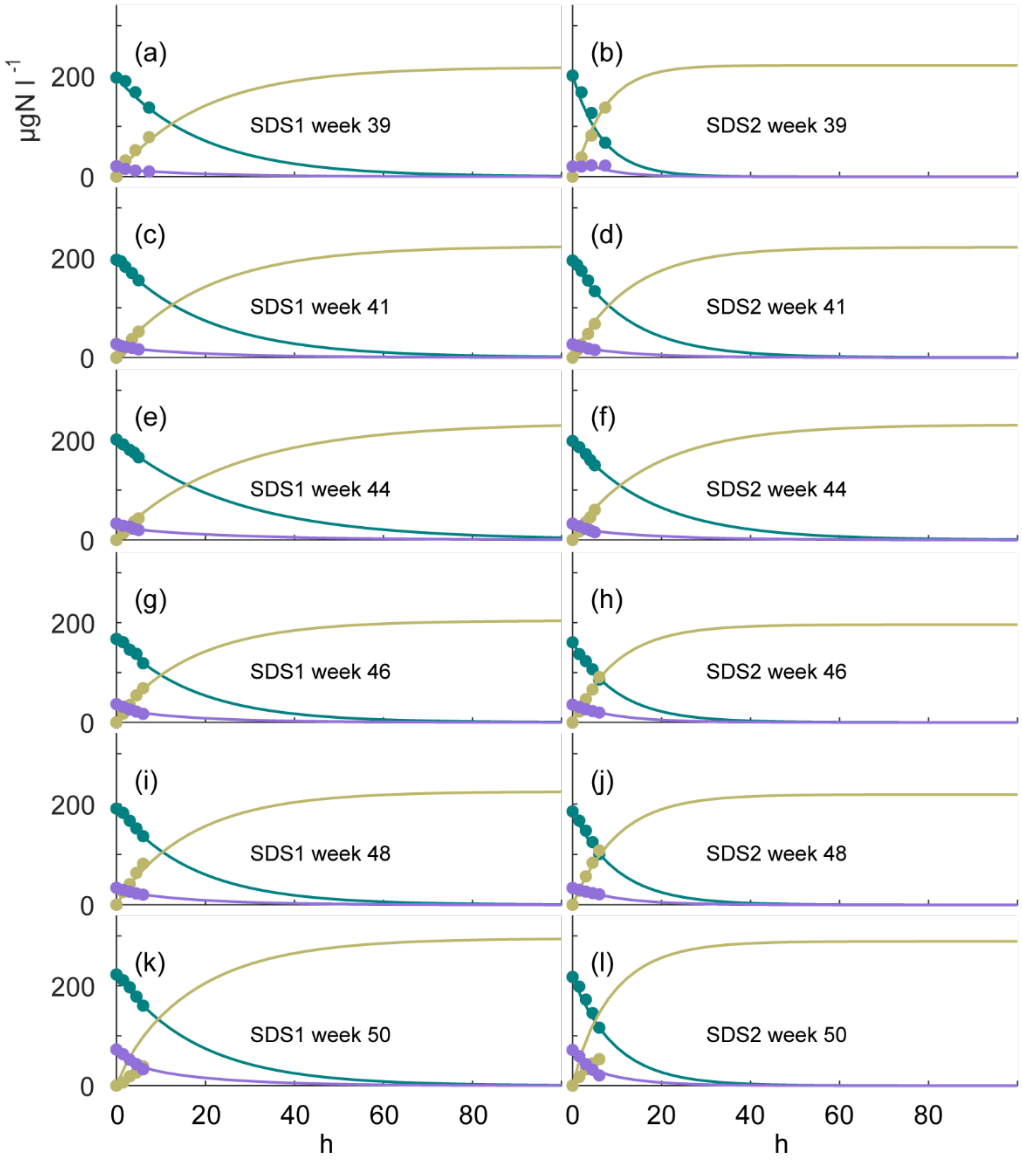

Figure S2. The data of the ammonium tests and the fitted pseudo-first order reaction rate models of the ammonium, nitrite and nitrate concentrations, with Reduced NOM. Teal dots: measured ammonium, teal line: modeled ammonium, purple dots: measured nitrite, purple line: modeled nitrite, dark yellow dots: measured nitrite increase, and dark yellow line: modeled nitrate increase.

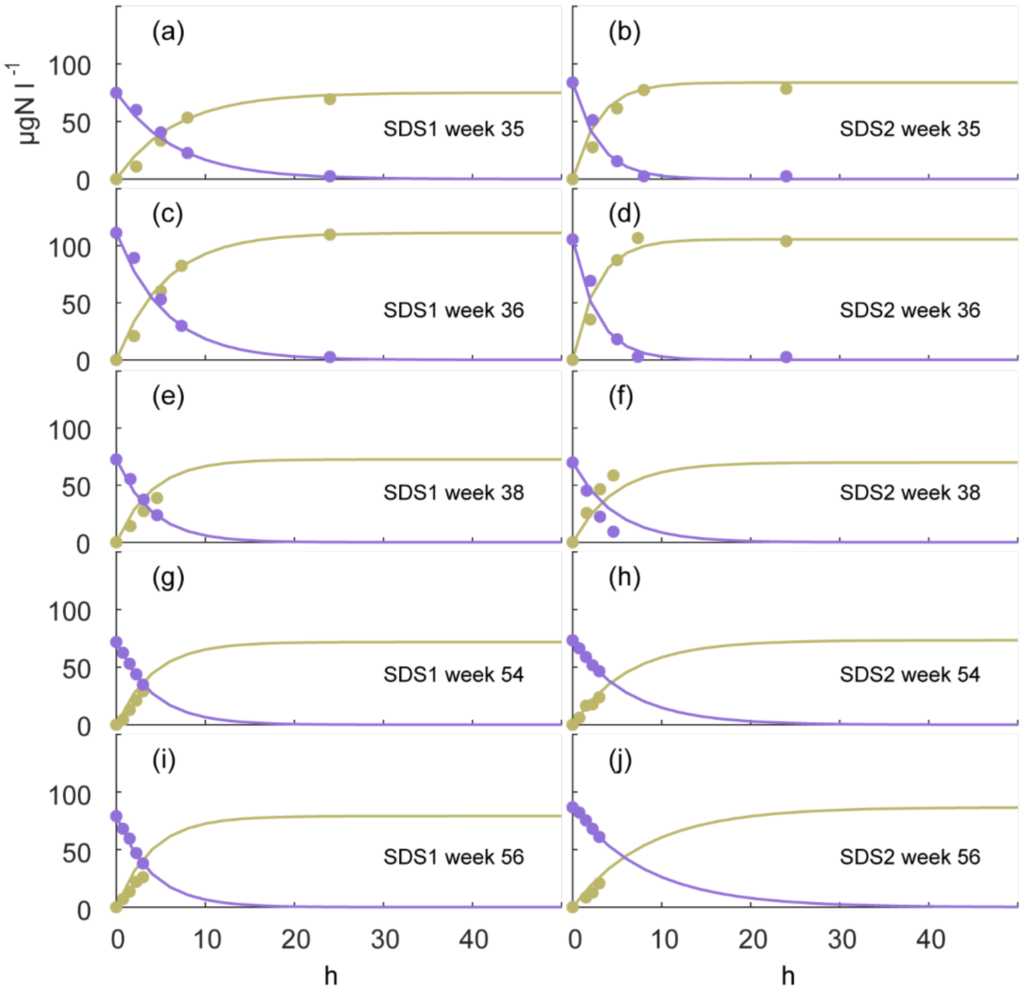

Figure S3. The data of the nitrite tests and the fitted pseudo-first order reaction rate models of the nitrite and nitrate concentrations, with Normal NOM. Purple dots: measured nitrite, purple line: modeled nitrite, dark yellow dots: measured nitrite increase, and dark yellow line: modeled nitrate increase.

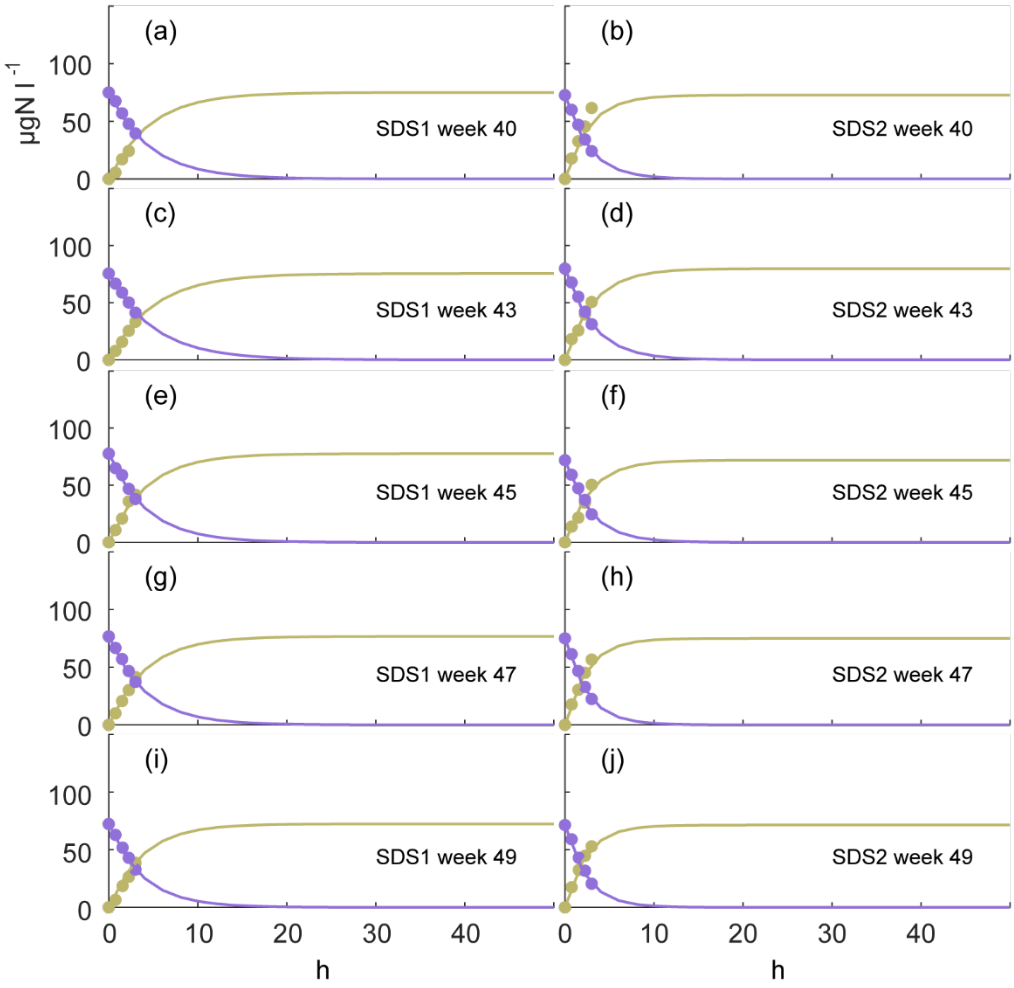

Figure S4. The data of the nitrite tests and the fitted pseudo-first order reaction rate models of the nitrite and nitrate concentrations, with Reduced NOM. Purple dots: measured nitrite, purple line: modeled nitrite, dark yellow dots: measured nitrite increase, and dark yellow line: modeled nitrate increase.

Section 5: The biofilm composition

The most abundant species in the combined samples of the biofilm from pipe loops SDS1 and SDS2 were the heterotrophic bacteria *Bradyrhizobium* sp. (25%) and *Reyranella soli* (15%). *Bradyrhizobium* sp. is capable of accumulating atmospheric nitrogen. Bacteria capable of denitrifying nitrite and nitrate were also present: *Rhodobacter* (7.6%), *Denitratisoma* (2.1%) and *Sterolibacterium* (1.9%). In addition, bacteria capable of the anammox reaction, *Gemmata* sp. (2.6%), were present in the biofilm.

Section 6: Interpreting the 3d image

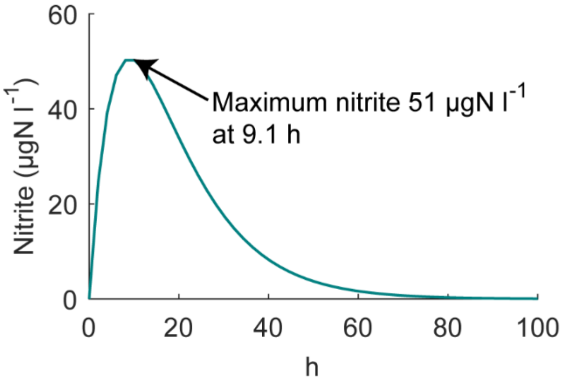

Figure S5. Example of the nitrite concentration change and the maximum value of nitrite at the point P (in Figure S6a-c). Each point of the surface ABCD in Figure S6, is a result of finding the maximum value of nitrite concentration with a pair of k_{NH4+} and k_{NO2-}.

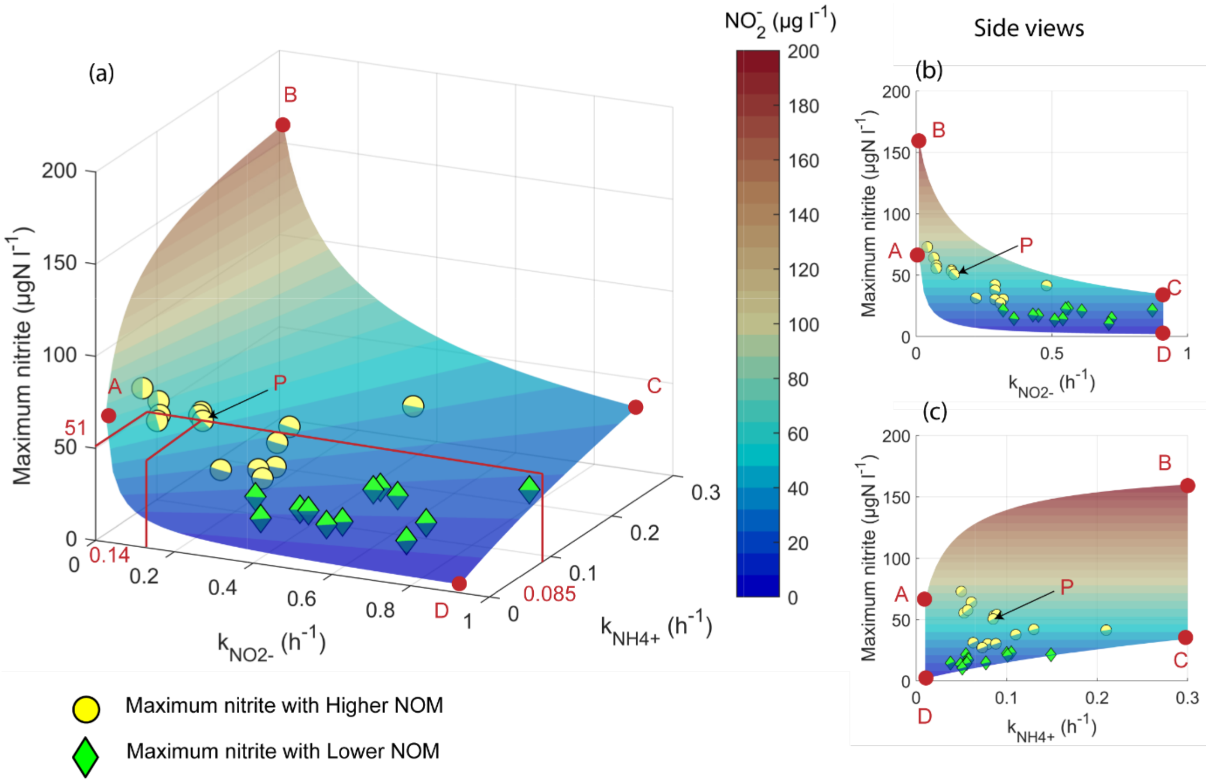

Figure S6. Reading the 3d image in more detail.

The surface in Figure 5 (main text) and Figure S6 is formed of the corners A, B, C and D. These are highlighted in the 3d view (S6a) and the side views (S6b and S6c). One of the points (P) is highlighted with an arrow, and in the 3d view the values on each axis are drawn as lines to the relevant axes. All the highlighted points are located on the surface, and the area of the point behind the surface is shadowed.

The curved surface of the maximum nitrite concentrations is formed from the maximum values of nitrite concentrations with each combination of k_{NH4+} and k_{NO2-}. An example of the curve is in Figure S5.

References

Hobbie, J.E., Daley, R.J., Jasper, S., 1977. Use of Nuclepore Filters for Counting Bacteria by Fluorescence Microscopy. Appl. Environ. Microb. 33, 1225-1228.

Kruglova, A., Gonzalez-Martinez, A., Kråkström, M., Mikola, A., Vahala, R., 2017. Bacterial diversity and population shifts driven by spotlight wastewater micropollutants in low-temperature highly nitrifying activated sludge. Sci. Total Environ. 605-606, 291-299. http://dx.doi.org/10.1016/j.scitotenv.2017.06.191.

Miettinen, I.T., Vartiainen, T., Martikainen, P.J., 1999. Determination of assimilable organic carbon in humus-rich drinking waters. Wat. Res. 33, 2277-2282. http://dx.doi.org/10.1016/S0043-1354(98)00461-8.

Van Der Kooij, D., Visser, A., Hijnen, W.A.M., 1982. Determining the Concentration of Easily Assimilable Organic Carbon in Drinking Water. J. Am. Water Works Ass. 74, 540-545.
